# Green Photocatalysis of Organic Pollutants by Bimetallic Zn-Zr Metal-Organic Framework Catalyst

**DOI:** 10.3389/fchem.2022.918941

**Published:** 2022-05-11

**Authors:** Xiaojuan Zhang, Rongfei Yu, Dandan Wang, Weihua Li, Yutao Zhang

**Affiliations:** ^1^ School of Chemistry and Chemical Engineering, Anshun University, Anshun, China; ^2^ University Rural Revitalization Research Center in Guizhou, Anshun, China; ^3^ Engineering Technology Center of Control and Remediation of Soil Contamination of Guizhou Science and Technology Department, Anshun, China

**Keywords:** metal-organic framework, green photocatalysis, dye wastewater treatment, rhodamine B, bimetallic

## Abstract

A series of bimetallic Zn-Zr metal-organic frameworks (Zn-Zr MOFs) with different Zn:Zr molar ratios has been synthesized *via* a green hydrothermal method. The structures and morphologies of these photocatalysts have been characterized and analyzed by FTIR, XRD, SEM, and nitrogen adsorption-desorption. The prepared Zn-Zr MOFs had large specific surface areas and pore volumes, favoring the adsorption of pollutant molecules, which in turn led to an improved photocatalytic effect. The photocatalytic activities of the Zn-Zr MOFs under visible light irradiation have been studied towards rhodamine B (RhB) as a target pollutant. The extent of degradation of RhB in a 40 mg/L aqueous solution reached 97.4%. The optimal photocatalyst could also degrade other dyes, suggesting a certain degree of universality.

## 1 Introduction

With the development of economy and society, and growing demand for industrial chemicals are accompanied by the increasing extent of environmental pollution. It has engendered an urgent need for the use of green catalytic methods to remove the organic molecules from industrial wastewater (Shanmuganathan et al., 2021). Among the various types of industrial wastewater, that from the printing and dyeing industries is some of the hardest to treat, being characterized by high volume, high content of organic pollutants, and high salinity (Kan et al., 2020). As the main pollutants in printing and dyeing wastewater, dyes are among the most important chemical species that need to be removed in wastewater treatment (Banerjee and Dastidar, 2005). Several dyeing wastewater treatment technologies have hitherto been devised, such as chemical oxidation, coagulation, and photocatalytic degradation, of which the latter is playing an increasingly important role in the field of water treatment (Xue and Zhu, 2019). The photocatalytic degradation of organic materials under light illumination has proven to be an efficient approach for wastewater treatment due to the simple treatment process, low cost, and environmental benignity (Yin et al., 2021). To date, ZnO, TiO_2_, SnO_2_, BiFeO_3_, and other photocatalysts have been designed and applied to degrade dye molecules in dyeing wastewater [such as rhodamine B (RhB), methyl orange (MO), Congo red (CR), acridine orange (AO), and methylene blue (MB)]. However, these photocatalysts have various disadvantages, such as fast recombination rates of photogenerated electrons and holes, relatively poor photocatalytic performance, low specific surface areas, and a tendency for agglomeration, which limit their practical application (Yang et al., 2021; Li et al., 2021).

Metal-organic frameworks (MOFs), as new crystalline porous materials, are attracting widespread attention in the field of photocatalysis due to their rich and tunable components, porous structures, large surface areas, uniform distributions of metal sites, and tunable optical absorption abilities (Zhao and Cai, 2021). Although single-metal MOFs have been reported to show a certain photocatalytic activity, they have poor photosensitivity and weak spectral absorption (Huang et al., 2021). Nevertheless, single-metal MOFs can be doped or composited with other metals to improve the photocatalytic activity. Baten et al. prepared MIL-53 (Fe) doped with Fe^2+^, which showed excellent photocatalytic activity for the degradation of MB (Baten et al., 2021). Wang et al. synthesized a highly efficient bifunctional Cu-MOF photocatalyst, over which MB was almost completely degraded within 5 h (Wang et al., 2019). In this study, Zn-Zr metal-organic frameworks (Zn-Zr MOFs) have been synthesized by a one-pot hydrothermal method. The structures and morphologies of Zn-Zr MOF photocatalysts with different Zn:Zr molar ratios have been studied. The photocatalytic properties of Rhodamine B (RhB) of Zn-Zr MOFs with different Zn:Zr molar ratios and single-metal MOFs have been compared and analyzed. In additon, the photocatalytic degradations of CR, NR, AO, MB, and MG over Zn-Zr MOFs-1 have been studied, with the aim of providing reference data for the industrial treatment of dyeing wastewater. The ultimate purpose of this research was to prepare photocatalysts through simple synthetic methods that were active under sunlight irradiation for the degradation of environmental pollutants. To this end, we have focused on the synthesis of bimetallic MOF composites, the photocatalytic performances which were superior to those of the single-metal MOFs.

## 2 Materials and Methods

### 2.1 Materials and Techniques

Zinc (II) nitrate hexahydrate (Zn(NO_3_)_2_ **·**6H_2_O), zirconium (IV) chloride (ZrCl_4_), terephthalic acid (H_2_BDC), Congo red (CR), acridine orange (AO), methylene blue (MB), and malachite green (MG) were obtained from Shanghai Aladdin Biochemical Technology Co., Ltd. Rhodamine B (RhB), neutral red (NR), N,N-dimethylformamide (DMF), and anhydrous ethanol were obtained from Chemical Reagent Co., Ltd. The above reagents were analytically pure. They were used without any further pretreatments or purifications. Deionized (DW, 18.25 MΩ) was used to prepare all solutions.

Powder X-ray diffraction (XRD) patterns of various samples were recorded on an automated X-ray diffractometer (XRD, Bruker D8 Advance, Germany) employing Cu-*K*_α_ radiation (*λ* = 1.54060 Å), and scanning the 2θ range 10–80°. The morphologies of the photocatalysts were examined by scanning electron microscopy (SEM, Hitachi SU 8100, Japan). The surface functional groups present on the samples were identified by Fourier-transform infrared (FTIR) spectroscopy (Perkin-Elmer 100, Shanghai). Parameters of the pore distribution and specific surface area were determined by the Brunauer-Emmett-Teller (BET) method by recording N_2_ adsorption–desorption isotherms at 77 K (Quadrasorb evo^™^, United States). Variations in solution dye concentrations were monitored using a UV/Vis spectrophotometer (UV-5200PC, Shanghai). The visible light source for photocatalytic degradation experiments was a xenon lamp (HSX-F300, Beijing).

### 2.2 Synthesis of Photocatalysts

Zn(NO_3_)_2_ **·**6H_2_O and ZrCl_4_ in Zn:Zr molar ratios of 1:1.5, 2:1, and 3:0.5 were dissolved in absolute ethanol (10 ml) to give solution A. H_2_BDC (0.66 g) was dissolved in absolute ethanol (10 ml) to give solution B. Solutions A and B were then simultaneously dropped into DMF (10 ml) under magnetic stirring. The mixture was stirred at room temperature for 1 h, and then transferred to an autoclave, which was maintained at 150°C for 6 h. After allowing the autoclave to cool to room temperature, the precipitate was collected by centrifugation. It was washed three times each with DMF and deionized water and dried at 60°C for 12 h to afford the Zn-Zr metal-organic framework (Zn-Zr MOF). For Zn:Zr molar ratios of 1:1.5, 2:1, and 3:0.5, the Zn-Zr MOFs are denoted as Zn-Zr MOF-1, Zn-Zr MOF-2, and Zn-Zr MOF-3, respectively. A Zr metal-organic framework without Zn (Zr MOF) and a Zn metal-organic framework without Zr (Zn MOF) were synthesized by the same method. All MOF materials were stored in a dry box.

### 2.3 Photocatalytic Activity Experiments

Rhodamine B (RhB) was used as the target pollutant for the investigation of photocatalytic activity. To evaluate the photocatalytic performances of the synthesized samples, they were deployed at 40 ppm in aliquots (50 ml) of aqueous RhB solution. Each mixture was stirred for 30 min in a darkroom to achieve adsorption-desorption equilibrium. It was then irradiated with simulated sunlight from a 300 W xenon lamp under continuous stirring.

At intervals of 15 min during the irradiation, the absorbance of RhB was recorded by a UV/Vis spectrophotometer. The efficiency of dye degradation was calculated according to the degradation percentage (Eq. 1):

$$\text{R}\text{h}\text{B}\left(\%\right)=\left(1-\text{C}/{\text{C}}_{0}\right)\times 100\%=\left(1-\text{A}/{\text{A}}_{0}\right)\times 100\%$$ (1)

where C_0_ is the initial concentration of the dye, A_0_ is the initial absorbance of the dye, and C and A are the concentration of the dye and the corresponding absorbance at time t.

## 3 Results and Discussion

### 3.1 Structural Characterization of Composite Materials

#### 3.1.1 FTIR Measurements

**FIGURE 1:**
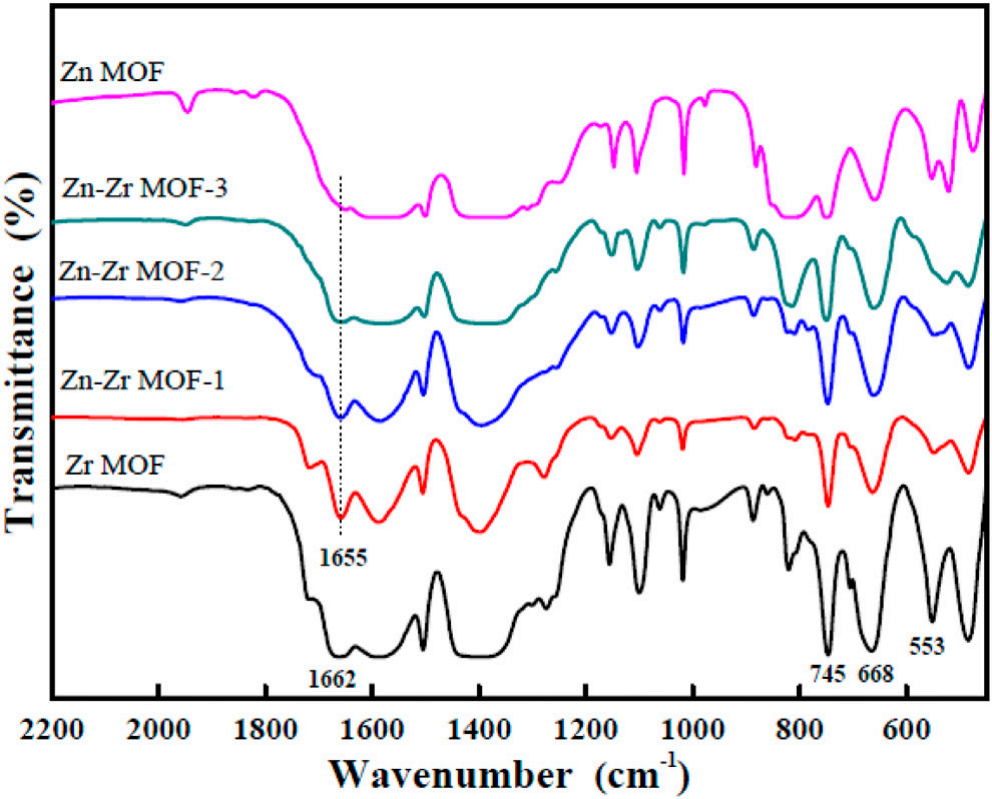
FTIR spectra of Zr MOF, Zn MOF, and Zn-Zr MOFs with different Zn:Zr molar ratios.

The FTIR spectra of Zr MOF, Zn MOF, and Zn-Zr MOFs with different Zn:Zr molar ratios are presented in Figure 1. It could be seen that the peak positions of each of the Zn-Zr MOF composites were consistent, and that the characteristic peaks of the Zr MOF and the Zn MOF were retained. This indicated the integrity and stability of the structures of the Zn-Zr MOF composites. The absorption bands in the range 1,200–600 cm^−1^ could be attributed to antisymmetric and symmetric stretching vibrations of the carboxylate groups of terephthalate. The bands at 745 cm^−1^ and 668 cm^−1^ could be ascribed to stretching vibrations of O-H and C-H bonds of terephthalate. The absorption peak at 553 cm^−1^ was a characteristic vibration peak of Zr-O. Notably, for the Zr MOF sample, the characteristic peak at 1,662 cm^−1^ could be attributed to the vibration absorption of the C=O bond in carboxylate, and this peak was shifted to lower wavenumber at 1,655 cm^−1^ when the second metal Zn was introduced into the MOF. This was consistent with Zn being coordinated by the carboxylate (Wang et al., 2021). Furthermore, the intensities of the characteristic absorption peaks of the Zr MOF were weakened, especially of that at 553 cm^−1^, when the molar proportion of Zn was increased, consistent with successful synthesis of the Zn-Zr MOF.

#### 3.1.2 Powder XRD Measurements

**FIGURE 2:**
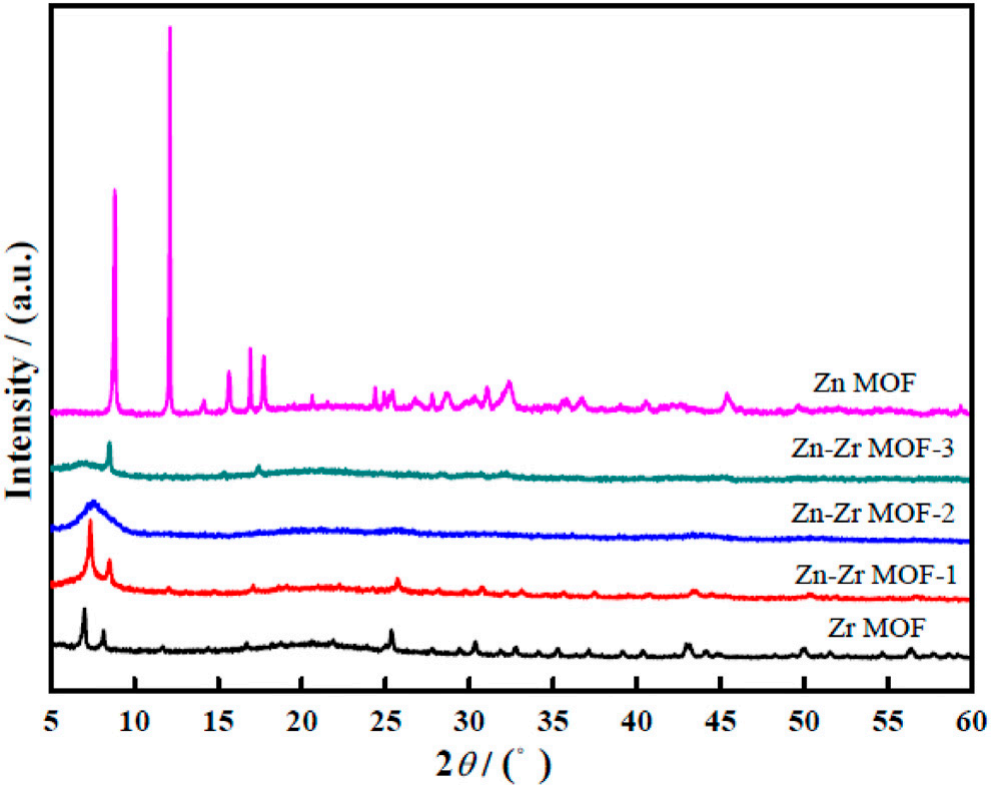
XRD patterns of Zr MOF, Zn MOF, and Zn-Zr MOFs with different Zn:Zr molar ratios.

Figure 2 presents the XRD patterns of Zr MOF, Zn MOF, and Zn-Zr MOFs with different Zn:Zr molar ratios. The peaks at 2θ = 7.0° and 8.2° could be indexed to the (111) and (002) crystal planes, respectively, of the Zr MOF phase structure (Kandiah et al., 2010). For Zn MOF, an obvious diffraction peak was seen at 2θ = 8.8°. The XRD patterns indicated that the synthesized Zr MOF and Zn MOF had good crystallinity. For Zn-Zr MOF-1, the characteristic diffraction peaks were observed at 2θ = 7.3° and 8.5°, slightly displaced from those of Zr MOF, but still clearly consistent. For Zn-Zr MOF-2, however, only a broad peak at 2θ = 7.3° was seen, and for Zn-Zr MOF-3 only the peak at 2θ = 8.5° was seen, with that at 2θ = 7.3° having disappeared. Evidently, the crystal structure of Zr MOF was gradually modified by the introduction of Zn (Chen et al., 2021). Compared with Zr MOF, the peaks of the Zn-Zr MOF composites were obviously weakened or disappeared, but there were no obvious new peaks that could be attributed to discrete Zn or Zr compounds. This implied the successful synthesis of Zn-Zr MOF composites with a uniform distribution of Zn and Zr. As would be expected, the XRD pattern of Zn-Zr MOF-1 most closely resembled that of Zr MOF.

#### 3.1.3 SEM Measurements

**FIGURE 3:**
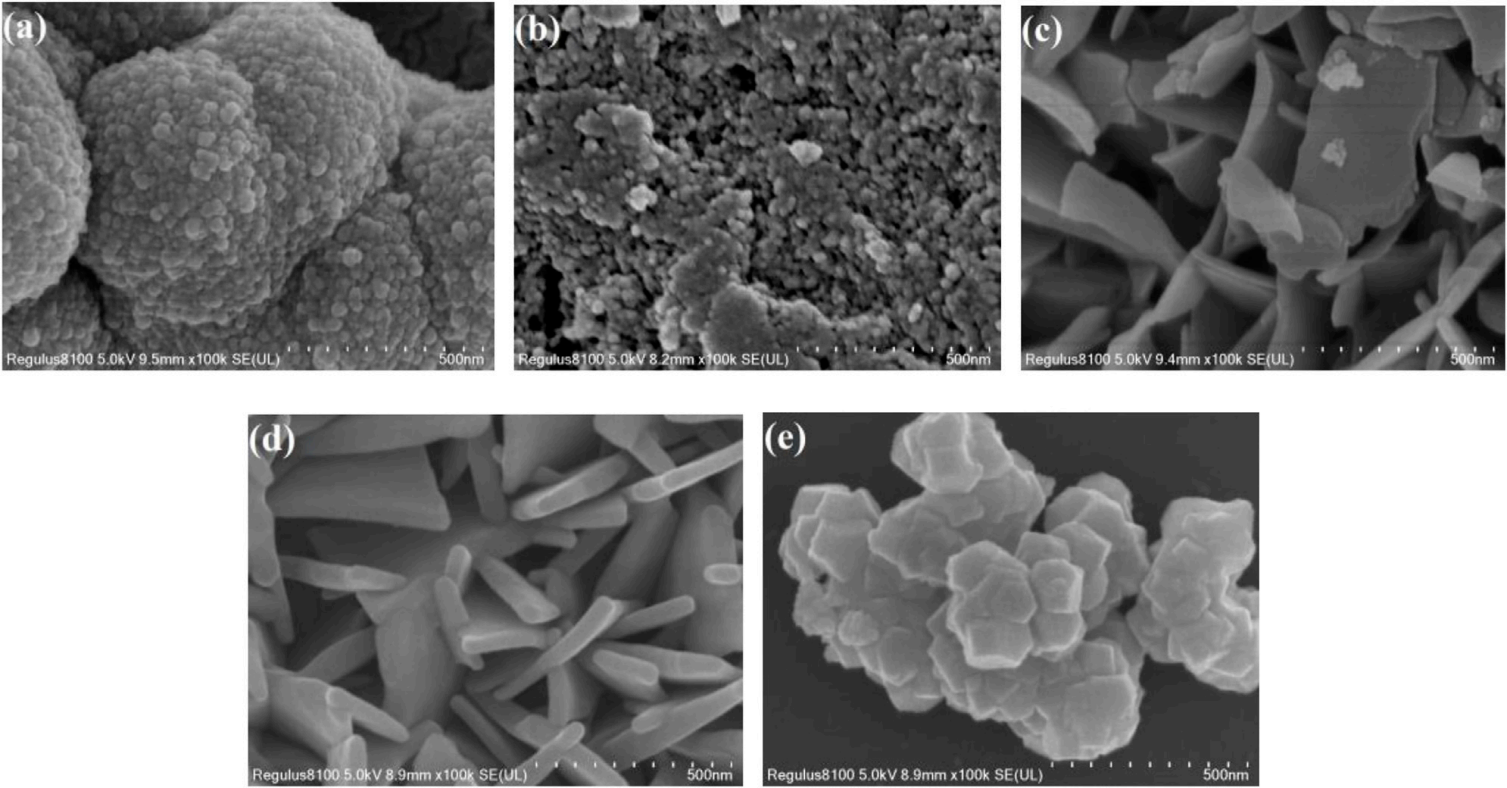
SEM images of Zn-Zr MOF-1 **(A)**, Zn-Zr MOF-2 **(B)**, Zn-Zr MOF-3 **(C)**, Zn MOF **(D)**, and Zr MOF **(E)**.

SEM images of Zr MOF, Zn MOF, and Zn-Zr MOFs with different Zn:Zr molar ratios are shown in Figure 3. As can be seen from Figure 3D, Zn MOF consisted of irregular blocks of random aggregates with smooth surfaces. Figure 3E shows that Zr MOF adopted a layered and lamellar structure with rough surfaces. As can be seen from Figures 3A–C, the morphologies and structures of the Zn-Zr MOFs with different Zn:Zr molar ratios progressively changed. Figure 3A shows that the structure of Zn-Zr MOF-1 consisted of small spheres made up of small particles of size about 100 nm. The SEM image of Zn-Zr MOF-2 (Figure 3B) revealed a structure comprising small particles, some of which aggregate into blocks. Figure 3C showed that Zn-Zr MOF-3 formed an irregular sheet structure with a smooth surface. It transpired that Zn-Zr MOF-2 and Zn-Zr MOF-3 could not efficiently photocatalyze the degradation of pollutants, because the surfaces of their structures were not sufficiently exposed. The SEM images revealed that the Zn:Zr molar ratio directly affected the assembly and hence the crystal structures of the Zn-Zr MOFs. This was corroborated by the N_2_ adsorption-desorption isotherms of the samples (*vide infra*) and the XRD measurements (*vide supra*). Based on the accumulated results, Zn-Zr MOF-1 was selected for physical adsorption studies.

#### 3.1.4 N_2_ Adsorption-Desorption Isotherms

**FIGURE 4:**
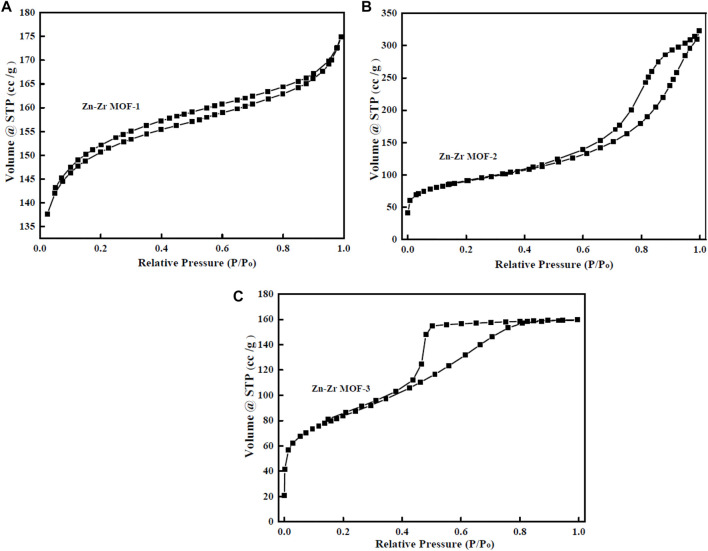
Nitrogen adsorption–desorption profiles of Zn-Zr MOF-1 **(A)**, Zn-Zr MOF-2 **(B)**, Zn-Zr MOF-3 **(C)**.

N_2_ adsorption-desorption was performed to further investigate the pore structure parameters of Zn-Zr MOFs with different Zn:Zr molar ratios. The results were presented in Figure 4 and Table 1. As could be seen in Figures 4A–C, the adsorption–desorption isotherms of the Zn-Zr MOF-1 sample showed type I/IV isotherms, indicative of microporous and mesoporous. For comparison, Zn-Zr MOF-2 and Zn-Zr MOF-2 samples revealed a type-IV shape pattern with an obvious hysteresis loop, which suggested that mesopores exist. As expected, from Table 1, the BET surface area and mean pore volume of Zn-Zr MOF-1 (460.4 m^2^/g, 0.27 cm^3^/g) were found to be larger than those of Zn-Zr MOF-2 (254.11 m^2^/g, 0.12 cm^3^/g) or Zn-Zr MOF-3 (294.94 m^2^/g, 0.24 cm^3^/g), indicating more pores in the former. Meanwhile, the average pore sizes of Zn-Zr MOF-1, Zn-Zr MOF-2, and Zn-Zr MOF-3 were evaluated as 2.35, 7.69, and 3.35 nm, respectively. The results showed that different Zn:Zr ratios modified the pores. The characterization data showed that larger specific surface area and pore volume increased the adsorption performance of the material, promoted contact between the pollutant and catalyst, and hence improved the catalytic efficiency. It was generally believed that the greater the surface area of a catalyst, the greater the number of active sites thereon (Wang et al., 2015), favoring molecular diffusion and binding of the reactant. Since the catalytic reaction was a surface-controlled process, the photocatalytic activity was improved and the separation efficiency of photogenerated electrons and holes was enhanced (Wu et al., 2021). Therefore, the structural characteristics of Zn-Zr MOF-1 may be envisaged as beneficial for the removal of organic pollutants from water.

**TABLE 1:** BET surface areas and total pore volumes of the synthesized samples.

**Sample**	**BET surface area (m^2^/g)**	**Pore volume (cm^3^/g)**	**Pore size (nm)**
Zn-Zr MOF-1	460.4	0.27	2.35
Zn-Zr MOF-2	254.1	0.12	7.69
Zn-Zr MOF-3	294.9	0.24	3.35

### 3.2 Photocatalytic Performance Study of the MOFs Towards RhB Degradation

**FIGURE 5:**
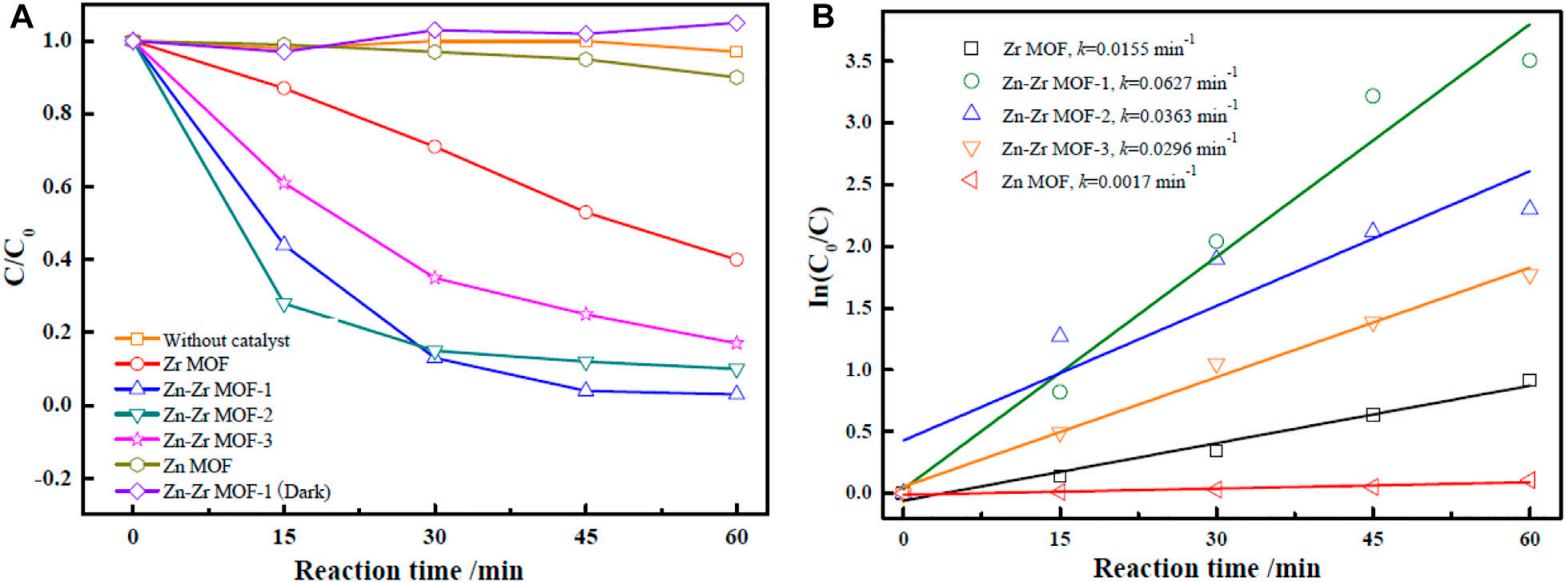
Photocatalytic degradation curves **(A)** and first-order kinetic degradation rates **(B)** of RhB with different catalysts under visible light irradiation.

The catalytic performances of the MOFs were evaluated by carrying out the photodegradation of RhB over Zr MOF, Zn MOF, and Zn-Zr MOFs. In order to eliminate the self-degradation effect of RhB in the photoreaction system, a blank degradation experiment on RhB was carried out under visible light irradiation without any catalyst. As could be observed from Figure 5, essentially no degradation of RhB occurred under visible light in the absence of a catalyst. Similarly, RhB showed almost no degradation in the presence of the Zn-Zr MOF catalyst but with the exclusion of light. As expected, the degradation of RhB increased with increasing reaction time in the presence of different photocatalysts under visible light irradiation. Zn MOF proved to be the least effective photocatalyst. The extent of degradation of RhB over Zn MOF at 0.6 g/L was just 10.3% after 60 min. This implied a high proclivity for electron-hole recombination under light irradiation, due to a slow rate of electron transfer and relatively poor absorption of visible light. Nevertheless, there was still a certain degree of degradation, mainly due to the photosensitivity of RhB (Liang et al., 2018). The photodegradation performance of Zr MOF was superior to that of Zn MOF, but inferior to those of the Zn-Zr MOFs, degrading 59.9% of RhB at a loading of 0.6 g/L within 60 min. The Zn-Zr MOF-1 composite showed the best RhB photodegradation performance, degrading 97.4% of RhB at a loading of 0.6 g/L within 60 min. The photocatalytic activities of the Zn-Zr MOFs with different Zn:Zr molar ratios were clearly higher than those of the single-metal MOFs, which may be attributed to some suppression of electron-hole recombination and improved absorption intensity in the visible light region (Li, 2021). However, it appeared that excessive Zn hinders the response of bimetallic Zn-Zr MOF catalysts to visible light. The photodegradation rates of RhB over Zr MOF, Zn MOF, and Zn-Zr MOFs with different Zn:Zr molar ratios were quantitatively evaluated using a kinetic rate model (Zhang et al., 2021), and the results were shown in Figure 5B and Table 2. The results of kinetic studies on the degradation of RhB over the different photocatalysts showed that the correlation coefficients were 0.8784, 0.9741, 0.9625, 0.8036, 0.9880 for linear plots of Zn MOF, Zr MOF, Zn-Zr MOF-1, and Zn-Zr MOF-2 and Zn-Zr MOF-3 respectively, indicating the process follows a pseudo-first-order model. The apparent rate constant *k* over Zn-Zr MOF-1 was 0.0627 min^−1^, around 4.0 times higher than that over Zr MOF and 36.9 times higher than that over Zn MOF. The apparent rate constant over Zn-Zr MOF-1 was about 1.7 times that over Zn-Zr MOF-2 and 2.1 times that over Zn-Zr MOF-3, which may be attributed to the abovementioned observations concerning the surface morphology and structure and the higher specific surface area and pore volume of Zn-Zr MOF-1.

**TABLE 2:** Kinetic parameters of the pseudo-first order for RhB degradation under visible light irradiation.

Dye	Sample	Pseudo-first order	
		*k*	*R* ^2^
RhB	Zn MOF	0.0017	0.8784
RhB	Zr MOF	0.0155	0.9741
RhB	Zn-Zr MOF-1	0.0627	0.9625
RhB	Zn-Zr MOF-2	0.0363	0.8036
RhB	Zn-Zr MOF-3	0.0296	0.9880

### 3.3 Effect of Photocatalyst Dosage on RhB Degradation

**FIGURE 6:**
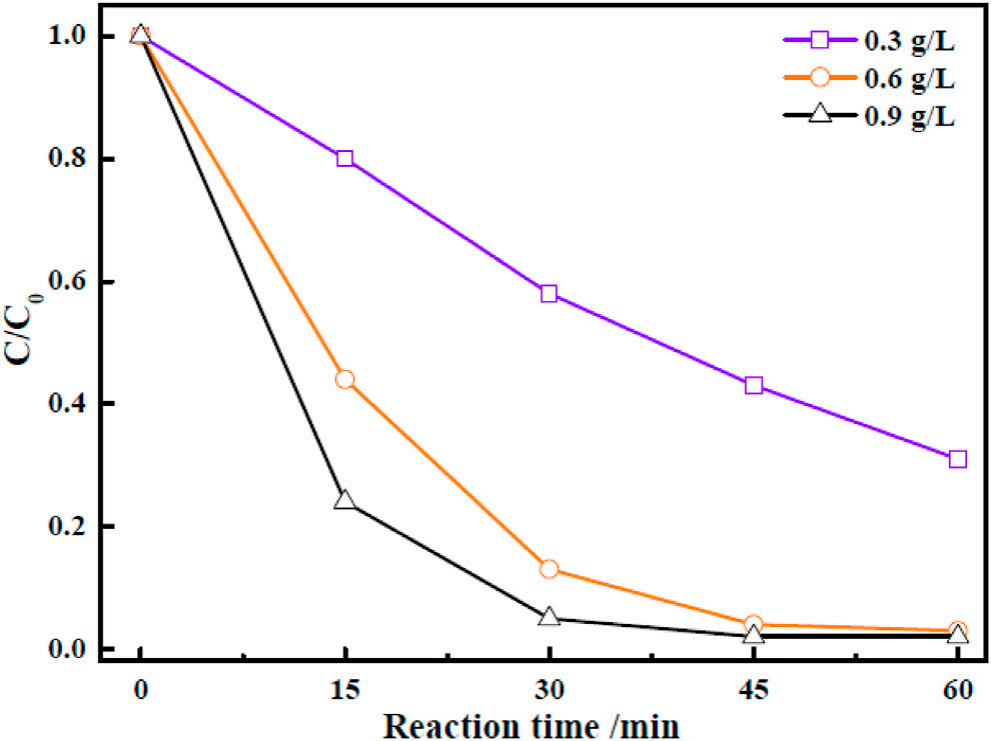
Effect of catalyst loading on the photocatalytic degradation of RhB under visible light irradiation.

In view of the results obtained in the preceding section, the Zn-Zr MOF-1 sample with Zn:Zr molar ratio 1:1.5 was selected to further investigate the effects of various factors on its photocatalytic activity. First, the effect of varying the loading amount of Zn-Zr MOF-1 photocatalyst in the RhB solution was investigated, keeping all other operating variables constant. The relevant results were displayed in Figure 6. It could be seen that the amount of degradation of RhB within 30 min increased with increasing loading of the photocatalyst, within a certain range, presumably due to the provision of more active sites. The extents of RhB degradation were 57.5, 97.4, and 98.1% after 60 min with Zn-Zr MOF-1 loadings of 0.3 g/L, 0.6 g/L, and 0.9 g/L, respectively. It could be observed from Figure 6 that there was no obvious change in the final degree of degradation of RhB when the catalyst loading was increased from 0.6 g/L to 0.9 g/L, which may possibly be attributed to a light shielding effect between particles (Liu et al., 2020). At the same time, a high catalyst loading in the degradation process would introduce a new environmental concern to the water supply ecology. Therefore, the Zn-Zr MOF-1 dosage was selected as 0.6 g/L.

### 3.4 Effect of the Initial RhB Concentration

**FIGURE 7:**
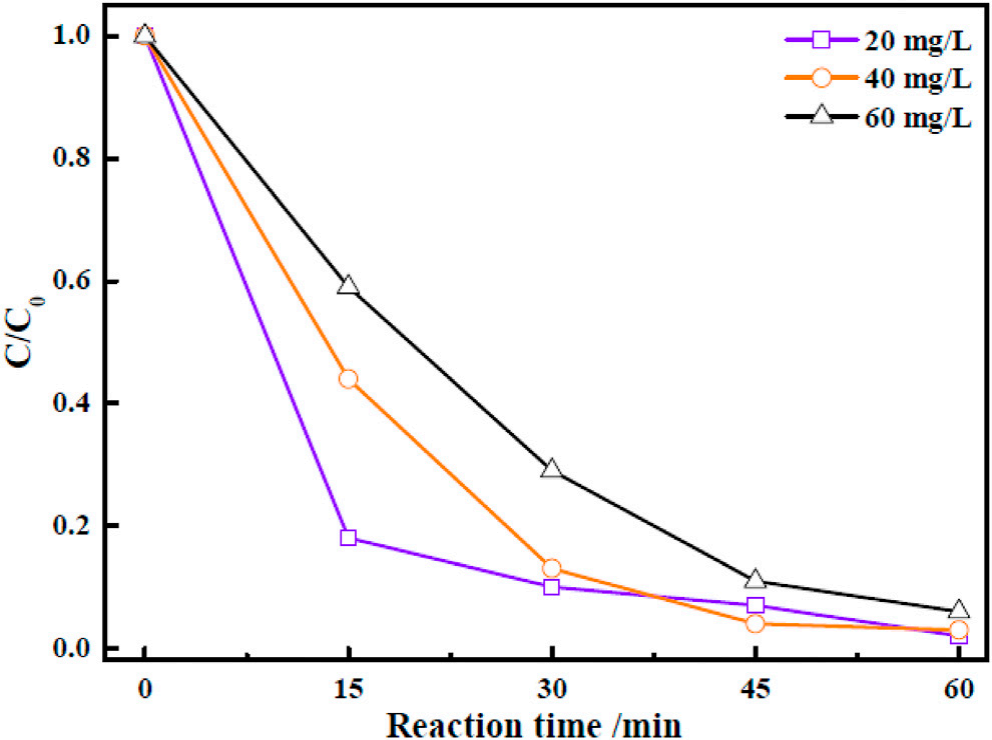
Effect of different concentrations of RhB on its photocatalytic degradation under visible light irradiation.

The effect of initial dye concentration on the photocatalytic performance of Zn-Zr MOF-1 catalyst was studied. In these experiments, other operating factors, such as the solution pH, light source, and catalyst loading, were all kept constant. The results of the assessment of the photocatalytic efficiency of Zn-Zr MOF-1 catalyst at various initial concentrations of RhB dye (20, 40, and 60 mg/L) were presented in Figure 7. It could be seen that the extent of photocatalytic degradation of RhB after 30 min decreased as its initial concentration was increased. The extent of photocatalytic degradation of an organic dye depended upon the available surface area of the photocatalyst, the production of various radicals, and the ability of these radicals to react with the dye molecules (Bagherzadeh et al., 2020) Here, an increase in the initial dye concentration reduced the mean length of the penetration path of incident photons in the solution. As a result, the likelihood of incident photons reaching the surface of the photocatalyst to produce electron–hole pairs was suppressed. After 60 min, the degrees of degradation of RhB at concentrations of 20 mg/L, 40 mg/L, and 60 mg/L were 98.1, 97.4, and 94.4%, respectively, indicating that Zn-Zr MOF-1 showed a good degradation effect towards RhB at different concentrations, and hence a certain practical application potential.

### 3.5 Reusability and Stability of Zn-Zr MOF-1 Composite

**FIGURE 8:**
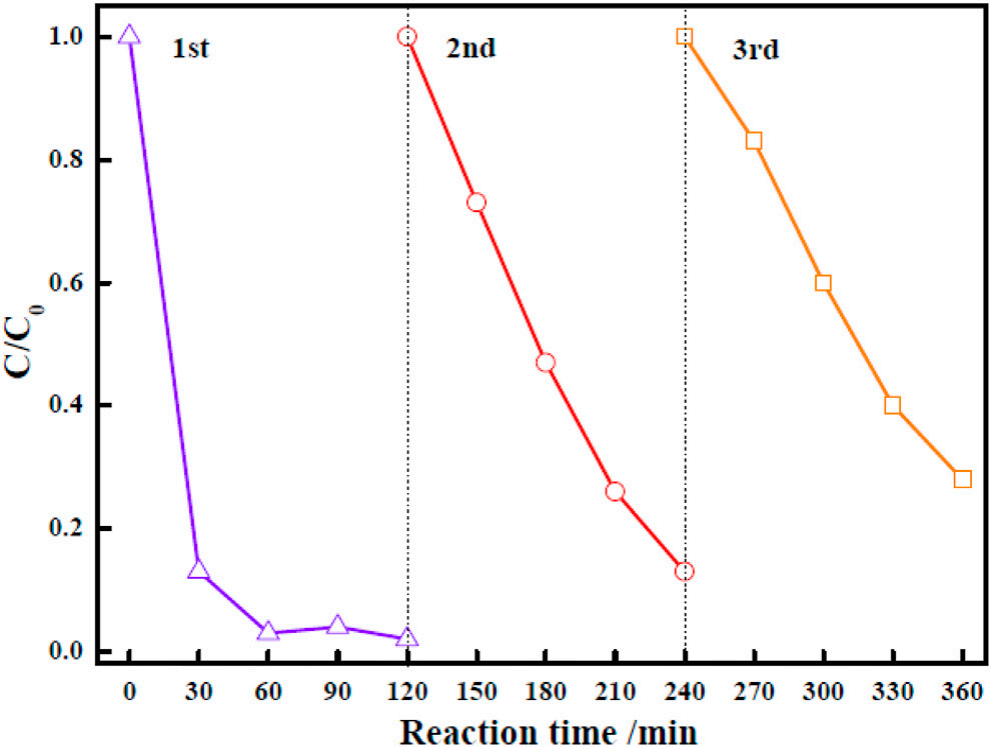
Recycling test of Zn-Zr MOF-1 in the photocatalytic degradation of RhB.

**FIGURE 9:**
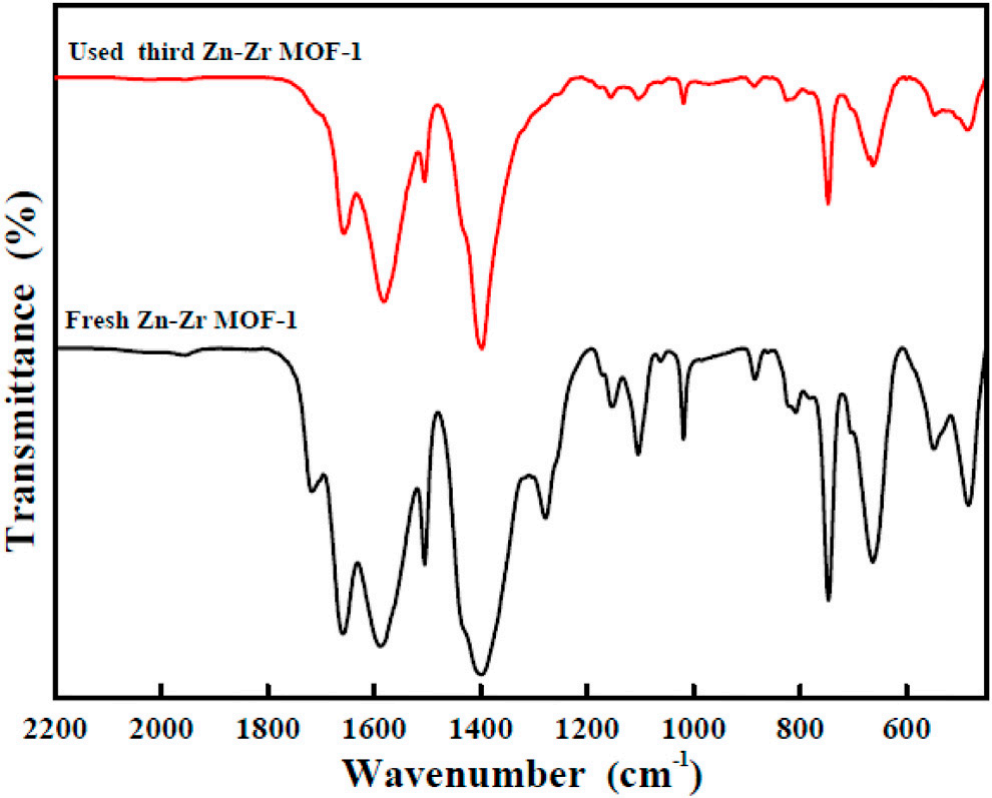
FTIR spectra of fresh and reused Zn-Zr MOF-1 catalyst.

For practical evaluation of the large-scale application of Zn-Zr MOF-1 photocatalyst, its reusability and stability in the photodegradation of RhB were investigated. Specifically, it was applied over three successive cycles. After each degradation process, the particles of the photocatalyst powder were separated. They were then collected from the reaction mixture by centrifugation, washed with ethanol and water, and oven-dried at 70°C. They were then applied in the next degradation cycle. The results of RhB degradation efficiency over Zn-Zr MOF-1 over three runs were presented in Figure 8. It could be seen that the degree of degradation of RhB was 97.7% in the first run, 86.8% in the second run, and 72.0% in the third run, indicating a loss of degradation efficiency over the three cycles. The decrease in RhB degradation efficiency in the course of reuse may be due in part to a small amount of catalyst being lost from the system during each recovery step (Duan et al., 2021). On the other hand, it may also be attributed to a loss of active sites on the catalyst surface. Pollutants or degradation products adsorbed on the material would gradually accumulate with increasing number of reaction cycles, progressively blocking more sites. Therefore, the relative reusability and stability of the Zn-Zr MOF-1 composite in photodegradation of the RhB was concluded. The FTIR spectra of Zn-Zr MOF-1 before and after three cycles of usage in the degradation of RhB were displayed in Figure 9. It could be seen that the spectrum remained essentially unchanged, albeit with a slight decrease in peak intensity. These results emphasized that a stable structure of the composite was maintained during the photocatalytic degradation process of RhB.

### 3.6 Photocatalytic Degradation of Other Dyes by Zn-Zr MOF-1

**FIGURE 10:**
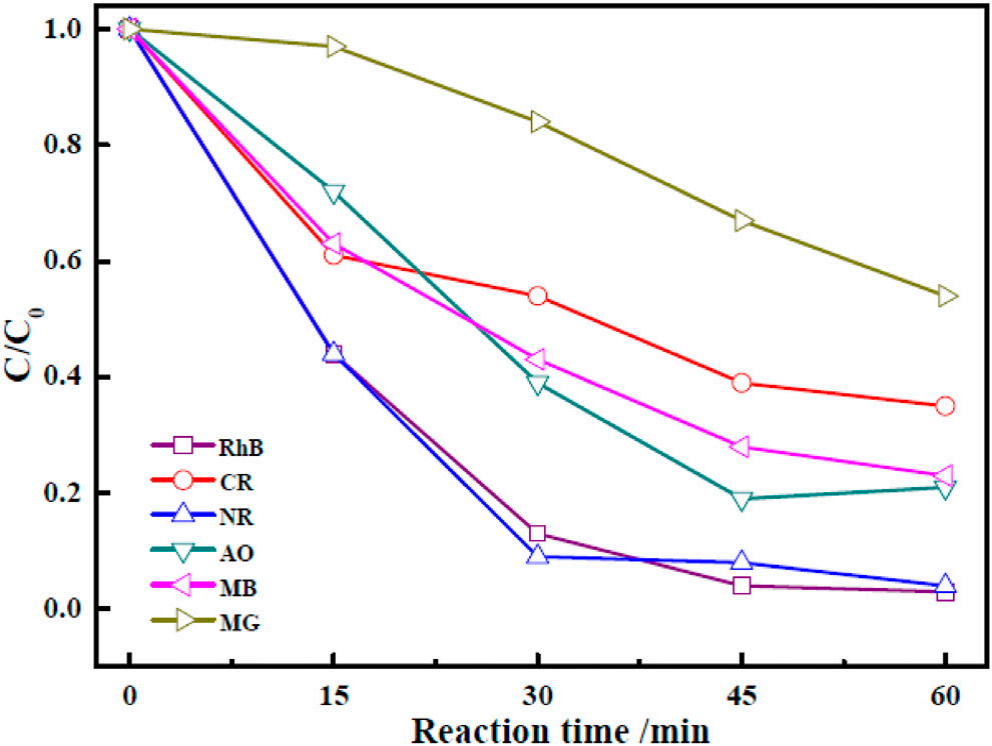
Photocatalytic degradation curves of different dyes over Zn-Zr MOF-1.

It was crucial to study the photocatalytic degradation of other organic dyes to assess the applicability of the photocatalyst. Therefore, photocatalytic degradations of CR, NR, AO, MB, and MG by Zn-Zr MOF-1 were studied, according to the optimum experimental conditions of catalytic degradation of RHB by Zn-Zr MOF-1, which the initial concentration of dye is 40 mg/L, the photocatalyst concentration is 0.6 g/L and the illumination time is 60 min and the results were shown in Figure 10. The photocatalytic degradation effect of Zn-Zr MOF-1 on NR was similar to that on RhB, reaching 95.9% after 60 min. Zn-Zr MOF-1 also showed good degradation effects towards AO and MB, amounting to 79.2 and 76.7%, respectively, after 60 min of illumination. However, the degrees of degradation of CR and MG were low, which may be related to the chemical structures of these dyes. Overall, the results demonstrated that the synthesized Zn-Zr MOF-1 photocatalyst could be used to degrade different organic dyes with reasonably good universality.

### 3.7 Photocatalytic Degradation RB Dyes by Different Photocatalysts As-Synthesized

The degradation percentage of dyes of RB by different photocatalysts under visible light listed in Table 3. As can be seen, the catalytic degradation rate of RHB by Zn-Zr MOF-1 synthesized in this paper was significantly higher than that of TiO_2,_ ZnTiO_3_ and ZnTiO_3_, which had comprehensive significant advantages in terms of catalyst dosage and catalytic degradation time. Comparing with ZnTiO_3_@S Cu MOF and SnO_2_/RCS, Zn-Zr MOF-1 also had certain advantages in the photocatalytic degradation effect of RHB, which mainly reflected in the characteristics of less catalyst dosage and shorter catalytic degradation time of Zn-Zr MOF-1.

**TABLE 3:** Degradation RB dyes by different photocatalysts as-synthesized.

Photocatalyst	Dye	Dye concentration	Photocatalyst concentration	Light source	Radiation time (min)	Degradation	Ref.
TiO_2_	RhB	—	40 mg/40 ml	Visible	300	62.20	Huerta-Flores et al. (2019)
ZnTiO_3_	RhB	0.1 mM	4 g/L	Visible	210	55.00	Meng et al. (2019)
ZnTiO_3_@TiO_2_	RhB	—	40 mg/40 ml	Visible	300	87.50	Huerta-Flores et al. (2019)
ZnTiO_3_@S	RhB	10 ppm	0.03 g/30 ml	Solar	180	94.74	Tavakoli-Azar et al. (2020)
Cu-MOF	RhB	—	15 mg/40 ml	Visible	300	94.50	Wang et al. (2019)
Sn O_2_/RCs	RhB	40 mg/L	0.9 g/L	Visible	120	95.56	Shi et al. (2021)
Zn-Zr MOF-1	RhB	40 mg/L	0.6 g/L	Visible	60	97.40	This work

## 4 Conclusion

A series of bimetallic Zn-Zr MOF photocatalysts has been synthesized by a hydrothermal method. Compared with the single-metal MOFs, the photocatalytic activity of bimetallic Zn-Zr MOF is significantly improved. The effects of different Zn:Zr molar ratios on the structures and morphologies of the MOFs have been studied. The results showed that when the Zn:Zr molar ratio was 1:1.5, the surface morphology and structure of the Zn-Zr MOF were relatively uniform, and it had a high specific surface area and large pore volume, providing more reactive sites for the substrate. Under visible light irradiation, the extent of degradation of RhB by Zn-Zr MOF-1 reached 97.4% within 60 min, and the photocatalyst also showed good photocatalytic degradation activity towards other dyes. The results of this study may provide reference data for industrial organic wastewater treatment.

## Data Availability

The original contributions presented in the study are included in the article/Supplementary Material, further inquiries can be directed to the corresponding author.
